# Catheter Duration Threshold and Risk Factors for Central Line-Associated Bloodstream Infections in a Tertiary ICU with Endemic Carbapenem Resistance: A Case–Control Study

**DOI:** 10.3390/antibiotics15040407

**Published:** 2026-04-17

**Authors:** Enes Dalmanoğlu, Mehmet Özgür Özhan, Bülent Atik, Tülin Akarsu Ayazoğlu

**Affiliations:** 1 Department of Infectious Diseases and Clinical Microbiology, Faculty of Medicine, Balıkesir University, 10145 Balıkesir, Türkiye; 2 Department of Anesthesiology and Reanimation, Faculty of Medicine, Balıkesir University, 10145 Balıkesir, Türkiye; metozhan2003@yahoo.com (M.Ö.Ö.); bulent_atik@yahoo.com (B.A.); 3 Department of Intensive Care Unit (ICU), Faculty of Medicine, Balıkesir University, 10145 Balıkesir, Türkiye; akarsu.dr@gmail.com

**Keywords:** central line-associated bloodstream infection, catheter duration, case–control study, survival analysis, ROC analysis, carbapenem resistance, *Acinetobacter baumannii*, intensive care unit, risk factors, attributable mortality

## Abstract

**Background/Objectives:** Central line-associated bloodstream infections (CLABSIs) remain a leading healthcare-associated infection in intensive care units (ICUs), yet independent risk factors and evidence-based catheter duration thresholds have not been defined through analytical study designs in settings with endemic multidrug-resistant organisms (MDROs). **Methods:** A retrospective case–control study was conducted in the ICU of a tertiary teaching university hospital in western Türkiye (January 2019–December 2024). Cases (n = 74) were patients with confirmed CLABSIs per CDC/NHSN criteria; controls (n = 148) were randomly selected central venous catheter (CVC)-bearing patients without CLABSIs. A reduced multivariate logistic regression model (seven variables; events-per-variable ratio 10.6) identified independent risk factors. **Results:** In multivariate analysis, catheter duration (adjusted OR: 1.19 per day; 95% CI: 1.13–1.24; *p* < 0.001), renal replacement therapy (aOR: 3.66; 95% CI: 1.68–7.95; *p* = 0.001), vasopressor support (aOR: 3.04; 95% CI: 1.50–6.17; *p* = 0.002), APACHE-II score (aOR: 1.07 per point; 95% CI: 1.02–1.11; *p* = 0.002), lower Glasgow Coma Scale (aOR: 0.86 per point; 95% CI: 0.78–0.94; *p* = 0.002), mechanical ventilation (aOR: 2.48; 95% CI: 1.24–4.95; *p* = 0.010), and total parenteral nutrition (aOR: 2.33; 95% CI: 1.12–4.86; *p* = 0.024) were independently associated with CLABSI. The model demonstrated good discrimination (C-statistic: 0.864) and calibration (Hosmer–Lemeshow *p* = 0.425). Kaplan–Meier analysis showed CLABSI-free survival declining from 98.9% at day 7 to 42.9% at day 21 (log-rank *p* < 0.001); these within-study estimates reflect relative risk patterns given the artificial 1:2 case-to-control ratio. Receiver operating characteristic (ROC) analysis identified day 13 as an exploratory optimal cutoff (AUC: 0.818; 95% CI: 0.762–0.874; sensitivity: 77.0%; specificity: 74.3%). CLABSI-attributable ICU mortality was 20.3% (47.3% vs. 27.0%; *p* = 0.004). Late-onset CLABSIs (>10 days) were dominated by Gram-negative pathogens (68.3%) versus 35.7% in early-onset infections (Fisher’s exact *p* = 0.012), with *Acinetobacter baumannii* as the predominant organism (27.0%; 83.3% carbapenem-resistant). **Conclusions:** Each additional catheter-day is independently associated with a 19% increment in CLABSI odds, with an exploratory critical threshold at day 13 beyond which enhanced surveillance measures should be considered, pending external validation.

## 1. Introduction

Intensive care units (ICUs) represent the epicenter of invasive device utilization, with central venous catheters (CVCs) placed in 48–80% of critically ill patients [1,2]. Central line-associated bloodstream infections (CLABSIs) constitute the most clinically significant complication of CVC use and remain a leading preventable healthcare-associated infection worldwide [3].

The clinical burden of CLABSIs is substantial. A recent meta-analysis by Elangovan et al., pooling 36 studies, reported a CLABSI-associated mortality odds ratio of 3.19 (95% CI: 2.44–4.16) with a mean excess hospital stay of 16.1 days [4]. In low- and middle-income countries, the International Nosocomial Infection Control Consortium (INICC) documented CLABSI rates of 4.55 per 1000 catheter-days—nearly sixfold higher than US rates—with mortality approaching 40% [5].

Evidence-based prevention bundles, as demonstrated in the Keystone ICU Project [6], have reduced CLABSI rates by up to 66% [6,7]. In Türkiye, national surveillance demonstrated a decline from 5.65 to 2.82 per 1000 catheter-days between 2008 and 2017 [8]. However, tertiary referral centers with endemic multidrug-resistant organisms (MDROs) continue to report elevated rates. Carbapenem-resistant *Acinetobacter baumannii* and *Klebsiella pneumoniae* dominate Turkish ICUs, with carbapenem resistance exceeding 80% for *A. baumannii* [9,10]. These organisms’ exceptional biofilm-forming capacity on catheter surfaces compromises standard prevention measures [11]. Furthermore, age-dependent dysregulation of innate immunity in elderly ICU populations amplifies susceptibility to severe infections and diminishes treatment response [12].

The time-dependent relationship between catheter duration and CLABSI risk has been recognized since Cobb et al.’s controlled trial [13]. However, critical gaps persist: most studies employ descriptive designs without control groups, precluding identification of independent risk factors; the optimal catheter duration threshold has not been determined through analytical methods such as receiver operating characteristic (ROC) analysis; CLABSI-attributable mortality has rarely been quantified using case–control comparisons in endemic MDRO settings; and temporal pathogen dynamics across catheterization periods remain underexplored. A meta-analysis of catheter insertion site and infection risk further demonstrated that site selection alone may be insufficient to mitigate CLABSI risk in high-acuity populations [14].

To address these gaps, we conducted a case–control study over a six-year period (January 2019–December 2024) in an ICU with endemic carbapenem resistance. The primary outcome was identification of independent risk factors for CLABSI. Secondary outcomes included time-dependent CLABSI-free survival, determination of an evidence-based catheter duration threshold, pathogen distribution across catheterization periods, and CLABSI-attributable mortality.

## 2. Results

### 2.1. Patient Flow and Baseline Characteristics

**Figure 1:**
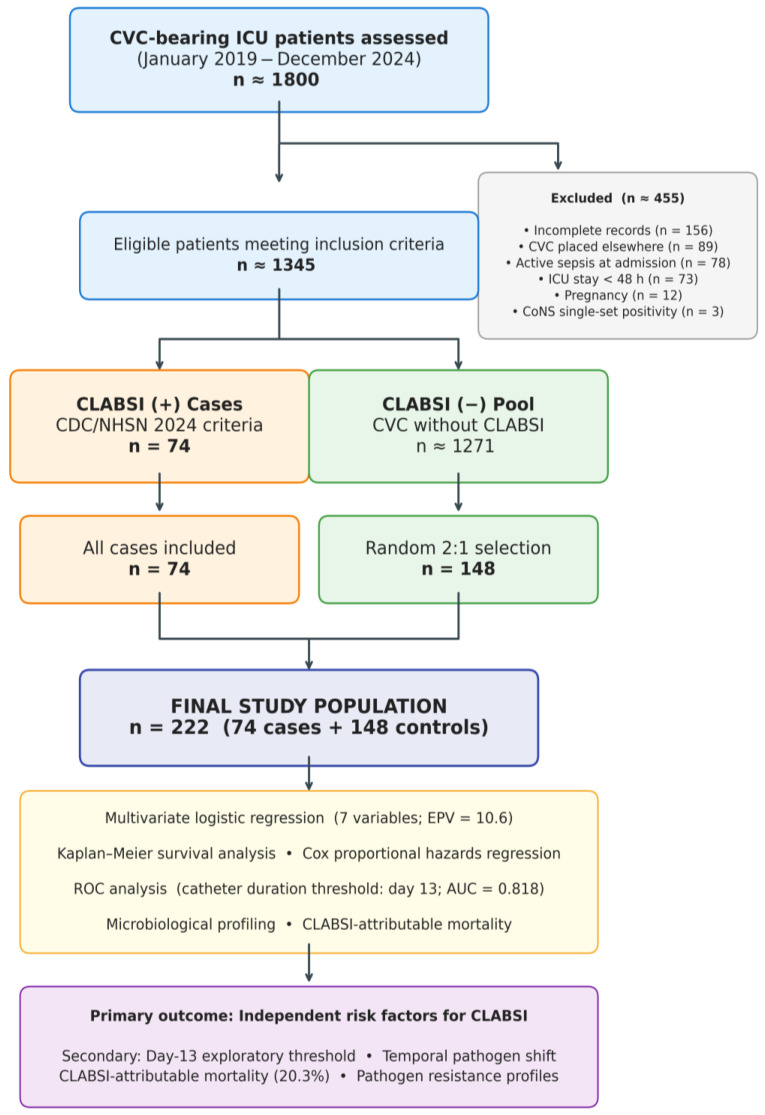
STROBE flow diagram of patient selection. Of approximately 1800 CVC-bearing patients, 455 were excluded. All 74 confirmed CLABSI cases were included; 148 controls were randomly selected at a 2:1 ratio.

During the six-year period, approximately 1800 patients had CVCs placed in the ICU. After applying exclusion criteria (Figure 1), 74 confirmed CLABSI cases and 148 randomly selected controls constituted the study population (n = 222). Three CoNS isolates with only single-set blood culture positivity were excluded as probable contaminants. No significant secular trend in CLABSI incidence was observed across the six study years (range: 10–14 cases/year; *p* for trend = 0.412). A post hoc comparison between pandemic years (2020–2021; n = 24 CLABSI cases) and non-pandemic years (2019, 2022–2024; n = 50 CLABSI cases) revealed no significant differences in annual CLABSI incidence (12.0 vs. 12.5 cases/year; *p* = 0.891), mean catheter duration (18.1 ± 6.8 vs. 17.5 ± 6.2 days; *p* = 0.714), APACHE-II scores (23.0 ± 8.1 vs. 22.1 ± 7.8; *p* = 0.652), or Gram-negative predominance (66.7% vs. 58.0%; Fisher’s exact *p* = 0.607).

Table 1 presents baseline characteristics with standardized mean differences. Most covariates demonstrated moderate balance (SMD 0.2–0.5), with the expected imbalances in catheter duration (SMD 1.12) and GCS (SMD 0.56)—both addressed through multivariate adjustment (Supplementary Table S1). Cases had significantly longer catheter durations (17.7 ± 6.4 vs. 10.4 ± 6.6 days; *p* < 0.001), higher vasopressor use (63.5% vs. 41.2%; *p* = 0.003), more RRT (35.1% vs. 20.3%; *p* = 0.025), and more TPN (40.5% vs. 24.3%; *p* = 0.019).

### 2.2. Independent Risk Factors for CLABSI

Table 2 presents the reduced seven-variable multivariate model. All seven variables were independently associated with CLABSI: catheter duration (aOR: 1.19 per day; 95% CI: 1.13–1.24; *p* < 0.001), RRT (aOR: 3.66; 95% CI: 1.68–7.95; *p* = 0.001), vasopressors (aOR: 3.04; 95% CI: 1.50–6.17; *p* = 0.002), APACHE-II (aOR: 1.07 per point; 95% CI: 1.02–1.11; *p* = 0.002), GCS (aOR: 0.86 per point; 95% CI: 0.78–0.94; *p* = 0.002), MV (aOR: 2.48; 95% CI: 1.24–4.95; *p* = 0.010), and TPN (aOR: 2.33; 95% CI: 1.12–4.86; *p* = 0.024).

**Figure 2:**
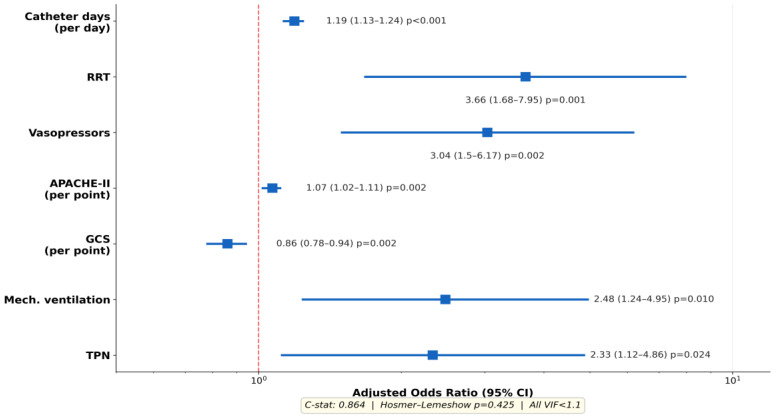
Forest plot of independent risk factors for CLABSI (reduced 7-variable model). C-statistic: 0.864; Hosmer-Lemeshow *p* = 0.425; all VIF < 1.1. All seven variables reached statistical significance (*p* < 0.05).

Model discrimination was excellent (C-statistic: 0.864), calibration was good (Hosmer–Lemeshow *p* = 0.425), and all VIF values were <1.1, confirming the absence of multicollinearity (Figure 2). While all seven variables reached statistical significance, internal validation through bootstrap resampling was not performed due to sample size constraints; the possibility of modest overfitting should be considered when interpreting individual odds ratios with wider confidence intervals. A sensitivity analysis including concurrent antibiotic use as an eighth covariate confirmed the robustness of the primary model (Supplementary Table S2). Catheter duration was the strongest modifiable risk factor, with each additional day associated with a 19% increase in CLABSI odds.

### 2.3. Kaplan–Meier and Cox Regression

**Figure 3:**
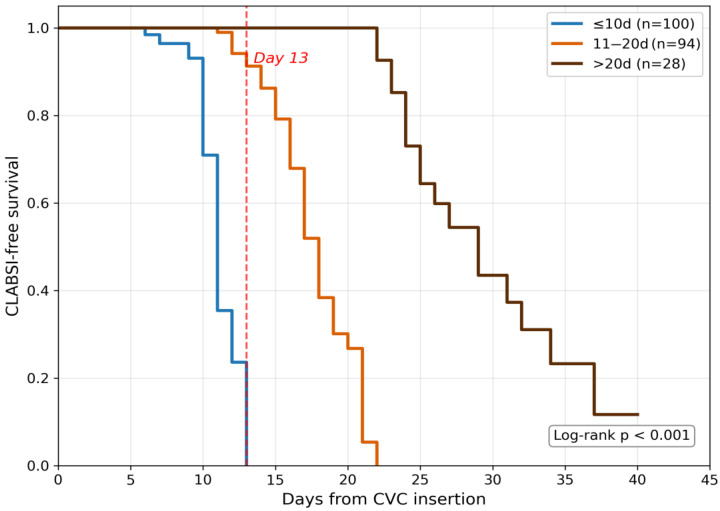
Kaplan–Meier CLABSI-free survival by catheter duration category. The vertical dashed red line indicates the ROC-derived day-13 threshold. Log-rank *p* < 0.001. Note: Absolute survival probabilities are inflated due to the artificial 1:2 case-to-control ratio and should be interpreted as relative between-group comparisons of temporal risk patterns rather than population-level event rates.

Kaplan–Meier analysis (Figure 3) demonstrated CLABSI-free survival rates of 98.9% (day 7), 81.0% (day 14), 42.9% (day 21), and 22.5% (day 28), with significant differences across duration categories: 14.0% event rate in the ≤10-day group (n = 100), 45.7% in the 11–20-day group (n = 94), and 60.7% in the >20-day group (n = 28) (log-rank *p* < 0.001). Given the artificial 1:2 case-to-control ratio, these survival estimates should be interpreted as illustrating relative temporal risk patterns rather than true population-level CLABSI probabilities.

Cox regression applied to cases only (n = 74; Table 3; C-index: 0.583) showed that none of the tested clinical covariates significantly predicted the speed of CLABSI onset (all *p* > 0.05), indicating that catheter duration itself remains the dominant temporal determinant within the range of covariates assessed. The low concordance index should be interpreted cautiously given limited statistical power with only 74 events. The proportional hazards assumption was verified (Schoenfeld global test *p* > 0.05 for all covariates).

### 2.4. Optimal Catheter Duration Threshold

**Figure 4:**
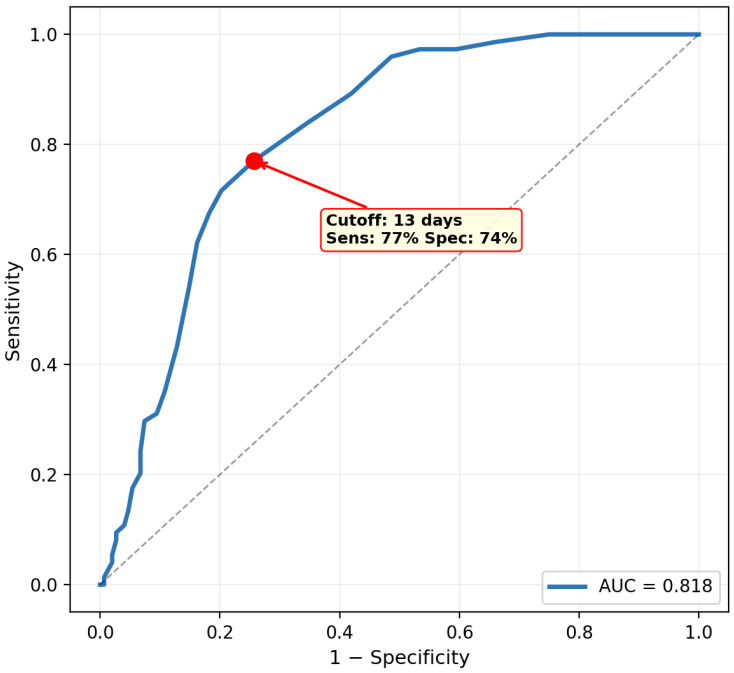
ROC curve for catheter duration as predictor of CLABSI (AUC: 0.818; 95% CI: 0.762–0.874; optimal cutoff: day 13; sensitivity 77.0%; specificity 74.3%; Youden index 0.514). AUC, sensitivity, and specificity are prevalence-independent. This threshold is exploratory and hypothesis-generating; external validation in prospective multicenter cohorts is required before clinical implementation.

ROC analysis (Figure 4) identified day 13 as an exploratory optimal cutoff (AUC: 0.818; 95% CI: 0.762–0.874; sensitivity: 77.0%; specificity: 74.3%; Youden index: 0.514). This threshold aligned with the Kaplan–Meier inflection point and represents the time point at which the balance between identifying true CLABSI cases and avoiding false positives is maximized. This finding should be considered exploratory and hypothesis-generating; prospective validation in multicenter cohorts is required before clinical implementation.

### 2.5. Microbiological Profile and Temporal Pathogen Dynamics

Table 4 details the pathogen distribution. Gram-negative organisms predominated (60.8%), followed by Gram-positive bacteria (16.2%), fungi (14.9%), and polymicrobial infections (8.1%). *Acinetobacter baumannii* was the most frequently isolated pathogen (27.0%; 83.3% carbapenem-resistant; 26.7% colistin-resistant among carbapenem-resistant isolates). *Klebsiella pneumoniae* accounted for 12.2% (53.8% carbapenem-resistant).

A significant temporal shift in pathogen distribution was observed: early-onset CLABSIs (≤10 days; n = 14) showed Gram-negative 35.7%, Gram-positive 35.7%, and fungi 28.6%; late-onset CLABSIs (>10 days; n = 60) showed Gram-negative 68.3%, fungi 26.7%, and Gram-positive 5.0% (Fisher’s exact test, *p* = 0.012).

### 2.6. CLABSI-Attributable Mortality

ICU mortality was significantly higher in cases (47.3% vs. 27.0%; OR: 2.42; 95% CI: 1.33–4.43; *p* = 0.004), yielding an attributable mortality of 20.3%. All extensively drug-resistant (XDR) *A. baumannii* infections were fatal (4/4, 100% vs. 44.3% for other pathogens; Fisher’s exact *p* = 0.039). Twenty-eight-day mortality similarly differed significantly (47.3% vs. 27.0%; *p* = 0.004).

## 3. Discussion

This case–control study is, to our knowledge, among the first to integrate multivariate risk factor analysis, survival analysis, and ROC-derived catheter duration thresholds in a setting with endemic carbapenem-resistant organisms. The study yields four principal findings.

First, catheter duration is independently associated with CLABSI risk (19% daily increment), consistent with but exceeding international estimates of 3–4% per day [15,16]. It is important to note that catheter duration, while classified as a modifiable risk factor, may also serve as a surrogate marker for overall illness severity, prolonged ICU stay, and cumulative exposure to nosocomial pathogens. The observed 19% daily risk increment represents a statistical association and should not be interpreted as a direct causal effect. Patients with longer catheter durations inherently have more severe illness trajectories, and the relationship between duration and CLABSI risk is likely bidirectional. Therefore, the day-13 threshold should be viewed as a risk-stratification tool for triggering enhanced surveillance rather than as a definitive indication for catheter removal [17]. The higher increment likely reflects the compounding effect of endemic MDROs with robust biofilm formation on catheter surfaces [11]. The ROC-derived day-13 threshold (AUC: 0.818) provides an exploratory clinical trigger not established in the prior CLABSI literature, which warrants validation in prospective, multicenter cohorts before incorporation into clinical practice guidelines. Current guidelines recommend daily catheter review without specifying duration-based escalation [7]. We propose that patients requiring CVCs beyond day 13 should receive enhanced measures: antimicrobial-impregnated catheter consideration [18], daily chlorhexidine bathing [19], antimicrobial lock therapy evaluation [20], and intensified microbiological surveillance.

Second, the 20.3% CLABSI-attributable mortality aligns with INICC and Turkish national data [5,8]. The International Society for Infectious Diseases 2024 position paper reported that CLABSI-associated mortality reached 39.8% in low- and middle-income countries, with an excess length of stay of approximately 17 days [21]. The 100% fatality in XDR *A. baumannii* infections underscores the therapeutic futility when prevention fails in this population [22,23].

It should be noted that the study period (2019–2024) encompasses the COVID-19 pandemic, during which CLABSI rates increased globally due to overwhelmed healthcare systems and reduced staffing ratios [24]. A meta-analysis demonstrated approximately 27% lower CLABSI odds in the pre-pandemic era [25], and an INICC study including Türkiye confirmed an increase in rates from 2.54 to 4.73 per 1000 catheter-days between 2019 and 2020 [26]. Although no significant differences were observed between pandemic and non-pandemic years in our cohort—consistent with the absence of a secular trend (*p* for trend = 0.412)—pandemic-related factors may have introduced unmeasured heterogeneity in infection control practices [24,25,26].

Third, the temporal pathogen shift from a balanced early-onset profile to Gram-negative dominance (68.3%) at >10 days carries empirical therapy implications. Early-onset CLABSIs likely reflect skin flora contamination during insertion [3], supporting vancomycin plus an echinocandin. Late-onset CLABSIs reflect intraluminal and environmental acquisition of endemic MDR organisms [11], supporting carbapenem-sparing regimens targeting *A. baumannii* and *K. pneumoniae* [27,28].

The colistin resistance rate of 26.7% among carbapenem-resistant *A. baumannii* isolates is particularly alarming, as colistin often represents the last-resort agent for extensively drug-resistant infections. This finding aligns with the global trend of increasing colistin resistance potentially involving chromosomal mutations and/or plasmid-mediated mechanisms, as has been documented across endemic settings in both high-income and low-to-middle-income countries [21,26]. The combination of carbapenem and colistin resistance effectively eliminates two of the three major therapeutic pillars, leaving only tigecycline-based combinations and newer agents such as ceftazidime–avibactam (which lacks activity against both metallo-β-lactamases and the OXA-type carbapenemases predominant in *A. baumannii*) or cefiderocol. These findings reinforce the urgency of prevention-focused strategies, as treatment options for late-onset CLABSIs caused by XDR *A. baumannii* are severely limited.

Fourth, the reduced seven-variable model addresses a methodological gap. Previous CLABSI studies, including Pitiriga et al. [29], have not reported comprehensive model diagnostics or EPV ratios. Our model’s C-statistic of 0.864 and Hosmer–Lemeshow *p* = 0.425 demonstrate robust discrimination and calibration, exceeding the methodological transparency of comparable studies.

From a practical standpoint, the day-13 threshold can be integrated into electronic health record systems as an automated alert, triggering a standardized reassessment protocol. This protocol could include mandatory daily catheter necessity review by a multidisciplinary team, consideration of catheter exchange over guidewire or de novo insertion at a new site, initiation of antimicrobial lock therapy for catheters that cannot be removed, and intensified microbiological surveillance with paired blood cultures. Such a structured approach would translate the statistical finding into a tangible clinical workflow, facilitating implementation across ICUs with varying resource levels. International validation of this threshold through multicenter prospective studies would strengthen the evidence base for guideline incorporation.

Furthermore, the reduced seven-variable model intentionally excluded diabetes mellitus and chronic pulmonary disease, which were significant in the initial nine-variable model but lost significance during model reduction. This likely reflects their collinearity with APACHE-II and SOFA scores, which capture the physiological impact of these comorbidities more comprehensively. A sensitivity analysis including these comorbidities yielded similar point estimates for the remaining covariates, supporting the robustness of the reduced model.

The independent associations with RRT (aOR: 3.66), vasopressors (aOR: 3.04), and MV (aOR: 2.48) confirm that multi-organ dysfunction synergistically amplifies CLABSI risk beyond catheter duration alone. TPN (aOR: 2.33) is a well-established risk factor for CLABSI through multiple mechanisms: lipid emulsions provide a nutrient-rich medium supporting microbial growth within catheter lumens, TPN administration requires frequent hub manipulation that increases contamination risk, and the hyperosmolar nature of TPN solutions may promote biofilm formation at the catheter tip [30,31]. A recent meta-analysis confirmed TPN, multilumen catheters, immunosuppression, and catheterization duration as independent CLABSI risk factors [32]. The exclusion of concurrent antibiotic use from the primary model was a deliberate analytical decision to avoid confounding by indication, as critically ill patients inherently receive more antibiotics; this variable’s strong univariate association (crude OR ~6.0) likely reflects disease severity rather than a biologically plausible independent pathway to CLABSI. Critically ill patients receive antibiotics because they are sicker, not the reverse; including this variable would have introduced confounding by indication, potentially attenuating the effect estimates of the true risk factors. A post hoc sensitivity analysis including concurrent antibiotic use as an eighth covariate yielded similar point estimates for all seven primary variables (all aORs within 10% of the primary model), while the antibiotic variable itself lost significance (*p* = 0.118), supporting its role as a confounder rather than an independent predictor.

This study has several strengths. To our knowledge, it is among the first to combine multivariate risk factor analysis, survival analysis, and ROC-derived catheter duration thresholds in a single case–control design within an endemic carbapenem-resistant setting. The comprehensive model diagnostics (C-statistic, Hosmer–Lemeshow test, VIF, EPV reporting) exceed the methodological transparency of most comparable studies. Furthermore, the temporal pathogen shift analysis and attributable mortality quantification provide clinically actionable data that extend beyond conventional descriptive surveillance.

### Limitations

Several limitations should be acknowledged. First, the retrospective single-center design limits generalizability. The study was conducted in a tertiary ICU with endemic carbapenem-resistant *A. baumannii* (>80% carbapenem resistance), which represents a high-risk setting not representative of all ICU environments. The day-13 catheter duration threshold was derived in this specific epidemiological context and may differ substantially in settings with lower MDRO prevalence, different prevention bundle adherence levels, different catheter types, or different patient demographics. Multicenter validation across diverse ICU settings is essential before this threshold can be recommended for broader clinical implementation. Second, individual patient-level bundle compliance data were unavailable; while quarterly audits suggested > 90% adherence, undetected variation in compliance may have influenced CLABSI rates. Third, the 222-patient sample (EPV 10.6) is adequate for the primary seven-variable model but limits statistical power for subgroup analyses and precludes internal validation through bootstrap resampling. Fourth, ROC-derived sensitivity and specificity, while prevalence-independent, would yield different predictive values in the general ICU population where CLABSI prevalence is substantially lower; the day-13 cutoff requires external validation, and this threshold may differ in ICU settings without endemic multidrug-resistant organisms, where biofilm-mediated catheter colonization dynamics and pathogen acquisition pressures differ substantially. Fifth, despite multivariate adjustment, residual confounding from unmeasured variables cannot be excluded, particularly given the substantial baseline imbalances (SMD > 0.5) in catheter duration (1.120), GCS (0.562), and concurrent antibiotic use (0.711); propensity score methods or instrumental variable approaches in larger cohorts may control for these imbalances more effectively. Sixth, all-cause rather than CLABSI-specific mortality was compared, as attributing mortality solely to CLABSIs in patients with multi-organ dysfunction is inherently challenging. Future multicenter prospective studies with embedded compliance monitoring, propensity score matching, and internal/external validation of the day-13 threshold are warranted.

## 4. Materials and Methods

### 4.1. Study Design, Setting, and Ethics

A retrospective case–control study was conducted in the ICU of a tertiary teaching university hospital in western Türkiye. The ICU is a closed unit. The study period encompassed 1 January 2019 through 31 December 2024.

Ethical approval was obtained from the Balıkesir University Non-Interventional Research Ethics Committee (decision No: 2025/176, date: 6 May 2025). Informed consent was waived given the retrospective design with anonymized data. The study adhered to the Declaration of Helsinki and STROBE guidelines for case–control studies [33].

### 4.2. Study Population and Sample Size

Cases were adults (≥18 years) with laboratory-confirmed CLABSIs per CDC/NHSN 2024 criteria [34]: a bloodstream infection in a patient with a CVC in place > 2 calendar days, not secondary to another infection site. For coagulase-negative staphylococci (CoNS), two positive blood culture sets from separate draws were required per NHSN criteria; isolates meeting only single-set criteria were excluded as probable contaminants.

Controls were randomly selected adult patients from the same ICU and period who had CVCs placed without developing CLABSIs, at a 2:1 ratio. Exclusion criteria (both groups): incomplete records, CVC placed at another facility, active sepsis at admission (Sepsis-3), pregnancy, ICU stay < 48 h.

Sample size justification: With 74 cases and a reduced 7-variable multivariate model, the events-per-variable (EPV) ratio was 10.6, exceeding the recommended EPV ≥ 10 threshold for stable logistic regression estimates [35].

### 4.3. Data Collection

Variables were extracted from electronic health records, ICU databases, and laboratory information systems (Figure 1).

Demographics and comorbidities included age, sex, body mass index (BMI), hypertension, cardiovascular disease, diabetes mellitus, chronic pulmonary disease, chronic kidney disease, malignancy, immunosuppression, cerebrovascular disease, chronic liver disease, and the Charlson Comorbidity Index (CCI) [36]. Clinical severity scores included APACHE-II, SOFA, and Glasgow Coma Scale (GCS) at ICU admission. CVC variables included insertion/removal dates, catheter-days, insertion site, type, lumen count, ultrasound guidance, and guidewire exchange. Treatment variables included mechanical ventilation (MV), vasopressors, renal replacement therapy (RRT), total parenteral nutrition (TPN), and antibiotic exposure. Infection data (cases only) included CLABSI date, time-to-CLABSI, pathogen, and resistance profile per Magiorakos et al. [37]. Outcomes included ICU/hospital length of stay, ICU/hospital mortality, and 28-day mortality.

### 4.4. CLABSI Prevention Bundle

A standardized five-component bundle per CDC/SHEA/IDSA guidelines [7] was institutional policy throughout: hand hygiene, maximal barrier precautions, chlorhexidine antisepsis, optimal site selection, and daily catheter necessity review. Quarterly audits conducted by the infection control team using direct observation checklists suggested > 90% compliance with insertion bundle components. However, individual patient-level compliance data—particularly for maintenance bundle elements such as daily necessity review and hub care—were not systematically recorded in the electronic health records during the study period. This is a recognized limitation common in retrospective infection control studies in Türkiye, where structured digital documentation of bundle adherence at the patient level has not been widely implemented [38]. The absence of patient-level data means that undetected variation in daily bundle adherence across shifts or clinical situations cannot be excluded and represents a potential source of residual confounding.

### 4.5. Microbiological Methods

Blood cultures were processed using the BD BACTEC™ FX system (Becton, Dickinson and Company, Sparks, MD, USA). Susceptibility testing was performed using the BD Phoenix™ M50 system (Becton, Dickinson and Company, Sparks, MD, USA) with EUCAST breakpoints [39]. Catheter tips were cultured using the semi-quantitative roll-plate method (≥15 CFU significant) [40].

### 4.6. Statistical Analysis

Continuous variables were expressed as means ± SD or medians (IQR). Categorical variables were presented as frequencies (%). Comparisons used the Mann–Whitney U test (continuous) and chi-square or Fisher’s exact test (categorical). Baseline balance was assessed using standardized mean differences (SMDs); SMD < 0.2 indicated good balance, 0.2–0.5 indicated moderate imbalance requiring adjustment [41]. Individual SMD values are reported in Supplementary Table S1.

Multivariate logistic regression: Variable selection followed a structured three-stage approach. First, all clinically relevant baseline and treatment variables were assessed in univariate analysis; variables with *p* < 0.25 entered the initial multivariate model. Second, an initial 9-variable model was constructed including catheter duration, APACHE-II, GCS, mechanical ventilation, vasopressor support, RRT, TPN, diabetes mellitus, and chronic pulmonary disease. Third, to achieve an EPV ratio ≥ 10 (with 74 events), the model was reduced from 9 to 7 variables (EPV = 10.6). Concurrent antibiotic use was excluded a priori due to confounding by indication. Model discrimination was assessed by the C-statistic, calibration by the Hosmer–Lemeshow test, and collinearity by variance inflation factors (VIF < 5 acceptable). CCI was excluded from the multivariate model due to collinearity with individual comorbidities. During model reduction from 9 to 7 variables to achieve EPV ≥ 10, diabetes mellitus and chronic pulmonary disease—which were significant in the initial 9-variable model (aOR: 4.71 and 4.08, respectively)—were removed as they lost significance in the presence of the composite illness severity markers (APACHE-II and SOFA). Results were reported as adjusted odds ratios (aORs) with 95% confidence intervals (CIs). Internal validation through bootstrap resampling or k-fold cross-validation was not performed due to sample size constraints (74 events). While the EPV ratio of 10.6 exceeds the recommended ≥ 10 threshold, the borderline nature of this ratio means that individual odds ratios—particularly those with wider confidence intervals—should be interpreted with caution, as modest overfitting cannot be excluded.

Kaplan–Meier analysis: CLABSI-free survival was estimated from CVC insertion. For cases, the event was CLABSI diagnosis; for controls, observations were censored at catheter removal or ICU discharge. The log-rank test compared survival curves across catheter duration categories (≤10, 11–20, and >20 days). For the temporal pathogen dynamics analysis, early-onset CLABSI was defined as infection occurring ≤10 days after CVC insertion, and late-onset CLABSI as infection occurring > 10 days after CVC insertion. In a case–control design, the case-to-control ratio (1:2) is artificial and does not reflect the true CLABSI prevalence; therefore, absolute survival probabilities derived from these curves are inflated relative to population-level estimates and are presented exclusively for between-group comparison of temporal risk patterns rather than as absolute event rates.

Cox proportional hazards regression: Applied to cases only (n = 74) to identify predictors of time-to-CLABSI onset, thus avoiding the methodological artifact of applying Cox to a case–control dataset where event status is predetermined. The proportional hazards assumption was verified by Schoenfeld residuals. Performance was assessed by the concordance index.

ROC analysis: Catheter duration (continuous, days) was assessed for its ability to discriminate CLABSI cases from controls. While the logistic regression model confirmed the independent significance of catheter duration, the ROC analysis serves a distinct complementary purpose, identifying the optimal duration cutoff that maximizes the balance between sensitivity and specificity. The optimal cutoff was determined using the Youden index (J = sensitivity + specificity − 1). AUC, sensitivity, and specificity are prevalence-independent metrics and remain valid in case–control designs; however, predictive values may differ in the general ICU population where CLABSI prevalence is lower.

Attributable mortality was calculated as the difference in ICU mortality between cases and controls. A post hoc sensitivity analysis was performed by adding concurrent antibiotic use as an eighth covariate to the primary logistic regression model to assess the robustness of the primary estimates. No correction for multiple comparisons was applied, consistent with standard practice in exploratory case–control studies; all *p*-values should be interpreted accordingly. Significance was set at two-sided *p* < 0.05. Analyses were performed using SPSS 29.0 (IBM Corporation, Armonk, NY, USA), R 4.4.0 (R Foundation for Statistical Computing, Vienna, Austria), and Python 3.12 (lifelines v0.28.0 and scikit-learn v1.4.2 packages).

## 5. Conclusions

Catheter duration is the strongest modifiable risk factor for CLABSI in a tertiary ICU with endemic carbapenem resistance, with a 19% daily increment in adjusted odds. ROC analysis identifies day 13 as an exploratory, hypothesis-generating threshold for intensifying surveillance—a clinically noteworthy finding that requires external validation in prospective multicenter studies before guideline incorporation. The 20.3% attributable mortality and the temporal shift from balanced to Gram-negative-dominant pathogen profiles further support differentiated empirical therapy strategies based on the timing of infection onset. These findings advocate for integrating duration-based escalation triggers into CLABSI prevention bundles, particularly where multidrug-resistant organisms are endemic.

## Figures and Tables

**Table 1:** Demographic and clinical characteristics of CLABSI cases and controls.

Variable	Cases (n = 74)	Controls (n = 148)	*p*	SMD
**Demographics**				
Age (years), mean ± SD	71.9 ± 13.2	68.6 ± 16.9	0.249	0.214
Male sex, n (%)	27 (36.5)	70 (47.3)	0.165	0.220
Female sex, n (%)	47 (63.5)	78 (52.7)	—	—
BMI (kg/m^2^), mean ± SD	28.3 ± 6.3	26.7 ± 5.0	0.058	0.214
**Comorbidities**				
Hypertension	32 (43.2)	62 (41.9)	0.962	0.026
Cardiovascular disease	24 (32.4)	45 (30.4)	0.878	0.043
Diabetes mellitus	22 (29.7)	31 (20.9)	0.200	0.202
Chronic pulmonary disease	21 (28.4)	26 (17.6)	0.092	0.262
Chronic kidney disease	9 (12.2)	26 (17.6)	0.397	0.153
Malignancy	11 (14.9)	21 (14.2)	1.000	0.020
Immunosuppression	12 (16.2)	13 (8.8)	0.154	0.225
CCI, mean ± SD	4.4 ± 1.9	4.0 ± 1.9	0.110	0.217
**Clinical Severity**				
APACHE-II, mean ± SD	22.4 ± 7.9	20.8 ± 6.8	0.159	0.228
SOFA, mean ± SD	8.8 ± 3.6	8.0 ± 3.5	0.095	0.233
GCS, mean ± SD	8.5 ± 3.6	10.5 ± 3.4	<0.001 *	0.562
**Catheter & Treatment**				
Catheter-days, mean ± SD	17.7 ± 6.4	10.4 ± 6.6	<0.001 *	1.120
Mechanical ventilation	52 (70.3)	83 (56.1)	0.058	0.297
Vasopressor support	47 (63.5)	61 (41.2)	0.003 *	0.458
Renal replacement therapy	26 (35.1)	30 (20.3)	0.025 *	0.337
Total parenteral nutrition	30 (40.5)	36 (24.3)	0.019 *	0.352
Concurrent antibiotic use	67 (90.5)	91 (61.5)	<0.001 *	0.711
**Outcomes**				
ICU LOS (days), mean ± SD	33.6 ± 12.3	18.6 ± 8.3	<0.001 *	1.432
ICU mortality	35 (47.3)	40 (27.0)	0.004 *	0.430
28-day mortality	35 (47.3)	40 (27.0)	0.004 *	0.430

* *p* < 0.05. SMD: standardized mean difference. Mann–Whitney U test was used for continuous variables; chi-square or Fisher’s exact test was used for categorical variables.

**Table 2:** Multivariate logistic regression: independent risk factors for CLABSI (reduced 7-variable model).

Variable	Crude OR (95% CI)	*p*	aOR (95% CI)	*p*	VIF
Catheter-days (per day)	1.17 (1.12–1.22)	<0.001 *	1.19 (1.13–1.24)	<0.001 *	1.03
APACHE-II (per point)	1.03 (1.00–1.06)	0.159	1.07 (1.02–1.11)	0.002 *	1.01
GCS (per point)	0.85 (0.79–0.92)	<0.001 *	0.86 (0.78–0.94)	0.002 *	1.06
Mechanical ventilation	1.85 (1.01–3.39)	0.058	2.48 (1.24–4.95)	0.010 *	1.03
Vasopressor support	2.48 (1.38–4.47)	0.003 *	3.04 (1.50–6.17)	0.002 *	1.05
Renal replacement therapy	2.13 (1.12–4.04)	0.025 *	3.66 (1.68–7.95)	0.001 *	1.03
Total parenteral nutrition	2.12 (1.15–3.91)	0.019 *	2.33 (1.12–4.86)	0.024 *	1.02

* *p* < 0.05. C-statistic: 0.864; Hosmer–Lemeshow *p* = 0.425; EPV: 10.6. All VIF < 1.1. Concurrent antibiotic use excluded a priori. Variables not reaching *p* < 0.25 in univariate analysis (including sex [*p* = 0.165], BMI, hypertension, cardiovascular disease, malignancy, and chronic kidney disease) were not entered into the multivariate model per the pre-specified selection criteria.

**Table 3:** Cox proportional hazards regression: predictors of time to CLABSI onset among confirmed cases (n = 74).

Variable	Hazard Ratio	95% CI	*p*
Age (per year)	0.994	0.969–1.020	0.671
Female sex	0.866	0.510–1.468	0.592
APACHE-II (per point)	1.007	0.977–1.038	0.650
CCI	1.044	0.879–1.239	0.626
Mechanical ventilation	0.612	0.353–1.060	0.080
TPN	0.995	0.594–1.667	0.986
RRT	1.257	0.719–2.198	0.423
Vasopressor support	1.173	0.697–1.973	0.548

C-index: 0.583 (limited discriminative ability with 74 events). Proportional hazards assumption verified (Schoenfeld global test *p* > 0.05 for all covariates). Interpretation: No tested clinical covariate significantly predicted the speed of CLABSI onset, suggesting that catheter duration itself is the dominant temporal determinant. The low C-index should be interpreted cautiously given limited statistical power.

**Table 4:** Distribution of CLABSI pathogens and antimicrobial resistance patterns (n = 74).

Pathogen	n	%	Resistance
**Gram-negative bacteria**	**45**	**60.8**	
*A. baumannii*	20	27.0	CR: 83.3% (15/18); Col-R: 26.7% (4/15)
*K. pneumoniae*	9	12.2	CR: 53.8% (7/13); ESBL: 22.2% (2/9)
*P. aeruginosa*	6	8.1	MDR: 50% (3/6)
*S. maltophilia*	6	8.1	Inherent MDR
Other GN	4	5.4	
**Gram-positive bacteria**	**12**	**16.2**	
*E. faecium*	4	5.4	VRE: 50% (2/4)
*E. faecalis*	4	5.4	VRE: 0% (0/4)
CoNS	2	2.7	MR: 100% (2/2)
*S. aureus*	2	2.7	MRSA: 50% (1/2)
**Fungi**	**11**	**14.9**	
*C. albicans*	6	8.1	
*C. parapsilosis*	3	4.1	
*N. glabratus* (*C. glabrata*)	2	2.7	
**Polymicrobial**	**6**	**8.1**	

CR: carbapenem-resistant; Col-R: colistin-resistant; ESBL: extended-spectrum β-lactamase; VRE: vancomycin-resistant enterococci; MRSA: methicillin-resistant *S. aureus*; MR: methicillin-resistant; CoNS: coagulase-negative staphylococci. Resistance percentages are calculated among isolates tested for the respective antimicrobial agent; denominators may differ from the total isolate count when susceptibility testing was not performed for all isolates. For *A. baumannii*, 18 of 20 isolates underwent full antimicrobial susceptibility testing (AST) via the BD Phoenix™ M50 system; the remaining 2 isolates were identified from catheter tip cultures without standardized AST. Colistin susceptibility was determined by broth microdilution (EUCAST reference method) and performed only on carbapenem-resistant isolates (n = 15). For *K. pneumoniae*, n = 9 represents CLABSI episodes with *K. pneumoniae* as the primary causative pathogen; carbapenem resistance was assessed across all recovered *K. pneumoniae* isolates (n = 13), including co-isolates from polymicrobial episodes; ESBL screening was performed on primary episode isolates (n = 9).

## Data Availability

Available from the corresponding author on request. Not publicly available due to privacy restrictions.

## Supplementary Materials

The following supporting information can be downloaded at: https://www.mdpi.com/article/10.3390/antibiotics15040407/s1, Table S1: Standardized mean differences (SMDs) for baseline covariates between CLABSI cases and controls; Table S2: Sensitivity analysis comparing the primary 7-variable model with an 8-variable model including concurrent antibiotic use.

## Abbreviations

CLABSI: Central line-associated bloodstream infection; CVC: Central venous catheter; ICU: Intensive care unit; MDRO: Multidrug-resistant organism; MDR: Multidrug-resistant; XDR: Extensively drug-resistant; RRT: Renal replacement therapy; TPN: Total parenteral nutrition; MV: Mechanical ventilation; CCI: Charlson Comorbidity Index; APACHE-II: Acute Physiology and Chronic Health Evaluation II; SOFA: Sequential Organ Failure Assessment; GCS: Glasgow Coma Scale; CoNS: Coagulase-negative staphylococci; EUCAST: European Committee on Antimicrobial Susceptibility Testing; ROC: Receiver operating characteristic; AUC: Area under the curve; SMD: Standardized mean difference; EPV: Events per variable; VIF: Variance inflation factor; OR: Odds ratio; aOR: Adjusted odds ratio; CI: Confidence interval; ESBL: Extended-spectrum β-lactamase; VRE: Vancomycin-resistant enterococci; BMI: Body mass index.
