# Supplementary material for: Catheter Duration Threshold and Risk Factors for Central Line-Associated Bloodstream Infections in a Tertiary ICU with Endemic Carbapenem Resistance: A Case–Control Study
Source: Antibiotics (Basel). 2026 Apr 17;15(4):407. doi: 10.3390/antibiotics15040407 (PMC13113509; doi:10.3390/antibiotics15040407)
Supplement: Supplementary file 1 [file antibiotics-15-00407-s001.zip › antibiotics-4231191-supplementary/Supplementary_Table_S1.pdf]

Supplementary Table S1. Standardized mean differences (SMD) for baseline covariates between CLABSI cases and controls

Variable	Cases (n=74)	Controls (n=148)	SMD	Balance Assessment
Age (years)	71.9 ± 13.2	68.6 ± 16.9	0.214	Moderate
Female sex, n (%)	47 (63.5%)	78 (52.7%)	0.220	Moderate
Hypertension, n (%)	32 (43.2%)	62 (41.9%)	0.026	Good
Cardiovascular disease, n (%)	24 (32.4%)	45 (30.4%)	0.043	Good
Diabetes mellitus, n (%)	22 (29.7%)	31 (20.9%)	0.202	Moderate
Chronic pulmonary disease, n (%)	21 (28.4%)	26 (17.6%)	0.262	Moderate
Chronic kidney disease, n (%)	9 (12.2%)	26 (17.6%)	0.153	Good
Malignancy, n (%)	11 (14.9%)	21 (14.2%)	0.020	Good
Immunosuppression, n (%)	12 (16.2%)	13 (8.8%)	0.225	Moderate
Cerebrovascular disease, n (%)	12 (16.2%)	31 (20.9%)	0.121	Good
Chronic liver disease, n (%)	5 (6.8%)	8 (5.4%)	0.058	Good
Charlson Comorbidity Index	4.4 ± 1.9	4.0 ± 1.9	0.217	Moderate
APACHE-II score	22.4 ± 7.9	20.8 ± 6.8	0.228	Moderate
SOFA score	8.8 ± 3.6	8.0 ± 3.5	0.233	Moderate
GCS	8.5 ± 3.6	10.5 ± 3.4	**0.562**	**Imbalanced***
Catheter days	17.7 ± 6.4	10.4 ± 6.6	**1.120**	**Imbalanced***
Mechanical ventilation, n (%)	52 (70.3%)	83 (56.1%)	0.297	Moderate
Vasopressor support, n (%)	47 (63.5%)	61 (41.2%)	0.458	Moderate
Renal replacement therapy, n (%)	26 (35.1%)	30 (20.3%)	0.337	Moderate
Total parenteral nutrition, n (%)	30 (40.5%)	36 (24.3%)	0.352	Moderate
Prior antibiotic use, n (%)	44 (59.5%)	74 (50.0%)	0.191	Good
Concurrent antibiotic use, n (%)	67 (90.5%)	91 (61.5%)	**0.711**	**Imbalanced***

*SMD: standardized mean difference. Balance thresholds per Austin (2009): <0.2 good balance; 0.2–0.5 moderate imbalance (addressed through multivariate adjustment); >0.5 substantial imbalance requiring careful interpretation. *Variables with SMD >0.5 were included as covariates in the multivariate logistic regression model to control for confounding.*
