# Supplementary material for: Catheter Duration Threshold and Risk Factors for Central Line-Associated Bloodstream Infections in a Tertiary ICU with Endemic Carbapenem Resistance: A Case–Control Study
Source: Antibiotics (Basel). 2026 Apr 17;15(4):407. doi: 10.3390/antibiotics15040407 (PMC13113509; doi:10.3390/antibiotics15040407)
Supplement: Supplementary file 1 [file antibiotics-15-00407-s001.zip › antibiotics-4231191-supplementary/Supplementary_Table_S2.pdf]

Supplementary Table S2. Sensitivity analysis: comparison of the primary 7-variable model with an 8-variable model including concurrent antibiotic use.

Variable	Primary Model (7 variables) aOR (95% CI)	p	Sensitivity Model (8 variables) aOR (95% CI)	p	Change in aOR (%)
Catheter duration (per day)	1.19 (1.13–1.24)	**<0.001**	1.18 (1.12–1.24)	**<0.001**	−0.8
APACHE-II (per point)	1.07 (1.02–1.11)	**0.002**	1.06 (1.02–1.11)	**0.004**	−0.9
GCS (per point)	0.86 (0.78–0.94)	**0.002**	0.87 (0.79–0.95)	**0.003**	+1.2
Mechanical ventilation	2.48 (1.24–4.95)	**0.010**	2.39 (1.18–4.83)	**0.015**	−3.6
Vasopressor support	3.04 (1.50–6.17)	**0.002**	2.89 (1.41–5.94)	**0.004**	−4.9
Renal replacement therapy	3.66 (1.68–7.95)	**0.001**	3.51 (1.59–7.73)	**0.002**	−4.1
Total parenteral nutrition	2.33 (1.12–4.86)	**0.024**	2.21 (1.05–4.67)	**0.038**	−5.2
Concurrent antibiotic use	—	—	*2.14 (0.82–5.58)*	*0.118*	*N/A*
**C-statistic**	0.864		0.868		+0.5
**Hosmer–Lemeshow p**	0.425		0.389		

aOR: adjusted odds ratio; CI: confidence interval; GCS: Glasgow Coma Scale; APACHE-II: Acute Physiology and Chronic Health Evaluation II. Bold p-values indicate statistical significance (p < 0.05). The 8-variable sensitivity model includes concurrent antibiotic use as an additional covariate. All seven primary variables retained their statistical significance and directionality in the sensitivity model, with point estimates changing by <6%, confirming the robustness of the primary model. Concurrent antibiotic use did not reach independent significance (p = 0.118), supporting its exclusion from the primary model on confounding-by-indication grounds.
